# DustVeil: Label-Free Real-Time Detection of Airborne Coal-Mine Dust in Camera Streams via Physically-Grounded Multi-Cue Fusion and Knowledge Distillation

**DOI:** 10.3390/s26175635

**Published:** 2026-09-04

**Authors:** Ziming Huang, Yujia Wang, Kun Huang, Jianwei Yang, Zimo Fan, Xiaodong Sun, Tielin Zhao, Lei Ji, Tong Zhang, Fanglue Zhang

**Affiliations:** 1 CCTEG Coal Mining Research Institute, Beijing 100013, China; huangziming@tdkcsj.com (Z.H.); yangjianwei@tdkcsj.com (J.Y.); fanzimo2000@163.com (Z.F.); zhaotielin@tdkcsj.com (T.Z.); jilei@tdkcsj.com (L.J.); zhangtong@tdkcsj.com (T.Z.); 2 State Key Laboratory of Intelligent Coal Mining and Strata Control, Beijing 100013, China; 3 School of Engineering and Computer Science, Victoria University of Wellington, Wellington 6140, New Zealand; wyujia626@gmail.com (Y.W.); huangkun101230@gmail.com (K.H.); 4 School of Computer Science and Engineering, University of New South Wales (UNSW Sydney), Sydney, NSW 2052, Australia; fanglue.zhang@unsw.edu.au

**Keywords:** dust detection, underground coal mine, visual plume localization, unsupervised learning, label-free, dark channel prior, knowledge distillation, real-time, machine-vision sensors, water-spray specificity

## Abstract

Airborne dust plumes are difficult to localize in underground mine-face video because the scene is dark, illumination moves with machinery, and dust is confused with lamp bloom, reflective steel, and water-spray aerosol. We present DustVeil, a label-free two-stage system for image-space plume localization. Its software teacher combines background-referenced veiling (C1), local texture decay (C2), and absolute dark-channel response (C3) with a probabilistic soft-OR, then applies glare and chroma gates. The teacher returns a dimensionless response map in [0, 1], a binary plume mask and the corresponding image-area ratio; it does not estimate dust concentration, particle-size distribution, respirable exposure, or hazard categories. Teacher outputs from 342 frames in 114 clips/24 sessions supervise a 0.47 M parameter TinyU-Net. Evaluation uses a 144-image synthetic calibration set and a 72-frame real test set drawn from 72 clips in 18 sessions, with all roles separated at clip and session levels. Thresholds are selected only on synthetic masks and frozen before real scoring. After replacing per-image score normalization with fixed baseline-normal calibration and using reference implementations of the anomaly methods, DustVeil obtains IoU/F1 of 0.366/0.500 and the lowest clean-frame false-positive area (3.7% versus 13.9–59.9%). A separate water-spray set quantifies visual specificity. TinyU-Net runs at 610 FPS for network-only inference and 233 FPS aggregate in the measured six-stream decode-to-mask pipeline; optical flow is excluded from these figures. The validated scope is six fixed visible-light RGB cameras with camera-specific unlabelled calibration at one site, rather than concentration monitoring or camera-disjoint deployment.

## 1. Introduction

Airborne coal dust presents respiratory and explosibility hazards in underground mining [1,2,3], but the quantities required for exposure control—mass concentration, respirable fraction, and time-weighted exposure—must be measured with calibrated physical instruments [4]. The narrower aim of this study is to extract complementary spatial information from existing mine-face video: where a camera-visible plume appears, how much of the image it occupies, and how that visual region evolves. DustVeil therefore returns an image-space response map and mask, not a value in mg/m^3^, a particle-size estimate, or a safe/tolerable/dangerous decision.

This visual task remains difficult. Black coal offers little contrast, head-lamps and machine lights move, reflective equipment produces broad glare, and water spray creates an aerosol with a similar veiling appearance. Pixel-level labels are also scarce because the plume boundary is diffuse and the operational footage is restricted. A useful system must therefore be label-free, explicit about false responses, and fast enough for several fixed streams.

DustVeil addresses these requirements with a software teacher and a compact student. The teacher fuses three interpretable responses—background-referenced veiling, local texture decay, and absolute dark-channel elevation—then applies glare and chroma gates. Its outputs are distilled into TinyU-Net. A clip/session manifest separates calibration, pseudo-label training, validation, baseline fitting, synthetic threshold selection, and real testing; a separate spray-only evaluation tests a central visual confounder.

**Novelty and contributions.** The novelty is not the invention of dark-channel response, texture attenuation, background subtraction or distillation in isolation. It is the task-specific integration and validation of these established components for low-light underground plume localization:

- a three-cue soft-OR teacher with underground-specific glare and chroma suppression and explicitly defined continuous and binary outputs;
- a leakage-controlled calibration/evaluation protocol with synthetic-only frozen thresholds, session-clustered uncertainty and a separate water-spray specificity audit;
- distillation into a measured real-time student, accompanied by fair model-specific preprocessing, fixed score calibration for reference methods, and reproducible gate, temporal and timing scripts; and
- quantitative analyses of cue combinations, gate trade-offs, background drift, threshold sensitivity, camera scope, annotation reliability, and end-to-end throughput.

The remainder of the paper reviews optical and learning-based plume sensing (Section 2), defines the workflow, data, algorithms, and evaluation protocol (Section 3), reports the quantitative results (Section 4), and discusses scope and limitations (Section 5 and Section 6).

## 2. Related Work

### 2.1. Optical and Video-Based Aerosol Sensing

Classical smoke and dust detectors combine background change, edge or wavelet attenuation, color, and motion [5,6,7]. Atmospheric veiling and the dark channel provide related cues for suspended particles [8,9]. Recent visual smoke and fire studies extend these ideas with RGB deep networks, color–contour fusion, anchor-free detection, temporal change accumulation, thermal imaging in smoke, and detector-based fire recognition [10,11,12,13,14,15]. The underlying optics are not determined by concentration alone: scattering depends on particle diameter relative to visible wavelength, particle shape and refractive index, illumination geometry, and optical path length [16]. Mine dust is polydisperse, while an RGB camera integrates broadband path radiance over approximately the visible spectrum. Consequently, a visual mask cannot by itself recover a particle-size distribution or respirable mass concentration. Supervised outdoor dust segmentation has been correlated with physical monitors [17], and coal-mine video has been studied with frame-level anomaly scores [18]; DustVeil instead evaluates label-free pixel localization and water-spray response.

### 2.2. Underground Machine Vision

Underground monitoring uses fixed visible cameras for personnel, equipment, and process supervision [19]. Low illumination, non-uniform artificial light, suspended dust, and water mist have motivated mine-specific enhancement and dehazing methods [20,21,22]. Those methods usually treat haze as an impairment to remove. Here, the camera-visible plume is the target, which makes false responses on lamps, colored machinery, and spray central to the evaluation.

### 2.3. Label-Free Learning and Distillation

PaDiM, PatchCore, SimpleNet, EfficientAD, student–teacher feature matching, and reverse distillation learn normal appearance and localize deviations without target-class labels [23,24,25,26,27,28]; Anomalib provides a common implementation framework for several anomaly-detection families [29]. Promptable segmentation offers another label-free route [30,31,32]. The student comparison spans U-Net, DeepLabv3, MobileNetV3, SegFormer, and transformer-based visual representations [33,34,35,36,37]. Standard object detectors such as YOLO target bounded objects and do not directly resolve diffuse plume boundaries [38]. These families are useful references, but they are class-agnostic or designed for crisp objects. In this study, the training clips have no pixel-level dust masks; the physics-guided teacher supplies pseudo-labels, and knowledge distillation transfers them to a lightweight segmentation network [39]. The revised comparison uses fixed baseline-normal score calibration, reference implementations, and architecture-appropriate RGB preprocessing so that the reported differences are not artifacts of per-image normalization or incorrect input conventions.

## 3. Materials and Methods

### 3.1. System Workflow, Software Boundary, and Returned Outputs

**Figure 1:**
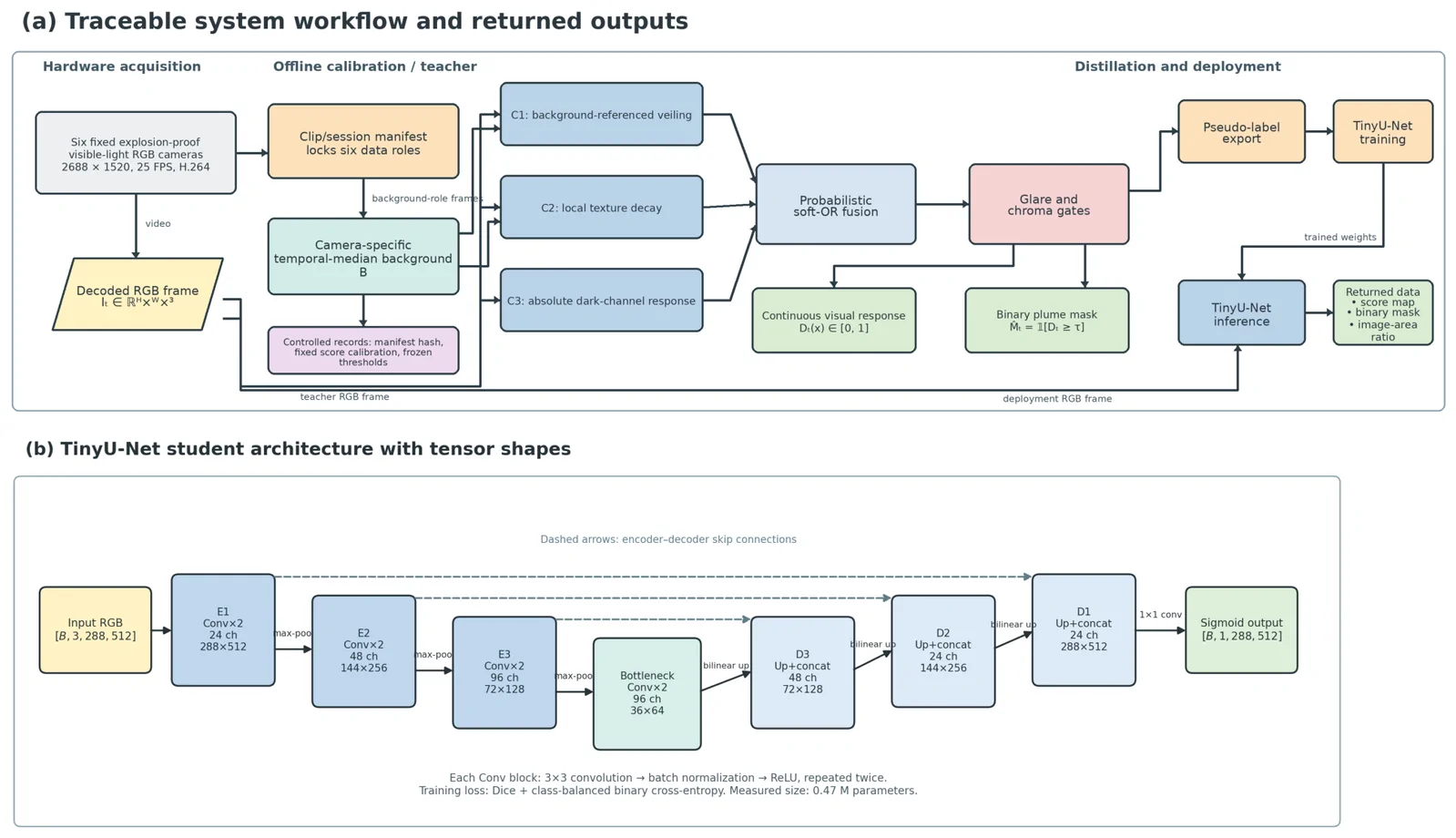
Traceable DustVeil workflow and TinyU-Net structure. (**a**) A visible-light RGB frame enters the offline teacher or deployed student. C1 is background-referenced veiling, C2 is local texture decay, and C3 is absolute dark-channel response; all are software-derived image maps. Fusion and gates yield a continuous visual response and binary plume mask. Solid arrows denote processing/data flow, and dashed arrows denote encoder–decoder skip connections. The teacher exports pseudo-labels, whereas deployment uses only TinyU-Net. (**b**) TinyU-Net encoder/decoder blocks, skip connections, and tensor shapes for input [B, 3, 288, 512] and output [B, 1, 288, 512].

Figure 1 presents the traceable workflow from acquisition to returned data and separates hardware, offline calibration, and deployment. The only sensing hardware used by DustVeil is the existing visible-light RGB camera. Decoding, background construction, C1–C3 computation, fusion, gates, pseudo-label export, training, and inference are software operations implemented with OpenCV 4.12.0.88, PyTorch 2.5.1, torchvision 0.20.1, NumPy 2.1.3, pandas 2.2.3, and SciPy 1.14.1 in a Python/CUDA workflow; these package versions are pinned in Supplementary Code S1. No ultraviolet, infrared, thermal, or particle-size detector is used. Controlled records comprise the clip/session manifest, baseline score-calibration parameters, frozen thresholds, configuration files, and audit hashes.

At teacher resolution, the decoded input has shape H×W×3=543×960×3. TinyU-Net receives a batched RGB tensor of shape B×3×288×512 and returns one sigmoid channel of shape B×1×288×512. The student probability map is resized to 960 × 543 before applying the frozen reporting threshold.

### 3.2. Data Acquisition and Background Model

The dataset was recorded with six fixed explosion-proof Hikvision color cameras mounted on powered supports at one longwall site. The exported streams are visible-light H.264 video at 2688 × 1520 pixels and 25 frames/s through integrated fisheye optics. They are decoded by OpenCV as BGR arrays and converted explicitly to RGB before all algorithms. No UV, IR, thermal, or active particle-sizing channel is present. The original six-role corpus contains 493 approximately 10 s clips from 107 global sessions; the held-out spray-specificity package is documented separately. A global session groups synchronized clips from one continuous operational episode, and a recording interruption longer than 60 s or an operational reset starts a new session.

The aerosol visible in these streams is a polydisperse mixture whose particles scatter broadband visible light. Image appearance depends on particle diameter relative to wavelength, refractive index, shape, path length, illumination, and camera geometry [16]. The present RGB workflow does not resolve individual particles and cannot separate the respirable fraction. This physical limitation motivates the output definition in Table 1: DustVeil localizes visual plume evidence only.

Let $I_{t}\in R^{H\times W\times 3}$ be an RGB frame and x∈Ω a pixel. For each camera, the clean reference *B* is the channel-wise temporal median of clip mid-frames assigned to the background role:

(1) $$B\left(x,c\right)={\mathrm{median}}_{t\in T_{B}}I_{t}\left(x,c\right),\;c\in \left\{R,G,B\right\}.$$

Here, $T_{B}$ contains only background-role observations. The median is intended to reject transient dust, personnel, machinery, and illumination when an uncontaminated view is present in more than half of the observations. Persistent occlusion, camera movement, and long-term drift violate this assumption and are quantified in the background audit. From *B*, we compute $B_{\min}\left(x\right)=\min_{c}B\left(x,c\right)$, luminance $B_{Y}=Y\left(B\right)$, the dark-background weight $g_{\mathrm{dark}}=\mathrm{clip}\left(\left(\tau_{\mathrm{dark}}-B_{Y}\right)/\tau_{\mathrm{dark}}\right)$, and local gradient energy $E_{B}=A_{r_{E}}(∥\nabla B_{Y}{∥}_{2})$.

### 3.3. Label-Free Teacher: Cues, Fusion and Gates

For each frame, we compute $I_{\min}\left(x\right)=\min_{c}I_{t}\left(x,c\right)$, luminance $I_{Y}=Y\left(I_{t}\right)$, and local gradient energy $E_{I}=A_{r_{E}}(∥\nabla I_{Y}{∥}_{2})$. The operator clip limits a value to [0, 1], $A_{r}$ is a square-window mean, and 1[·] is an indicator. The three software cues are:

(2) $$C1:\;v\left(x\right)=\mathrm{clip}\;\left(\frac{\left[I_{\min}\left(x\right)-B_{\min}\left(x\right)\right]\;g_{\mathrm{dark}}\left(x\right)}{s_{v}}\right),$$

(3) $$C2:\;t\left(x\right)=1\left[E_{B}\left(x\right)>\tau_{E}\right]\;\mathrm{clip}\;\left(1-\frac{E_{I}\left(x\right)}{E_{B}\left(x\right)+\epsilon_{t}}\right),$$

(4) $$C3:\;a\left(x\right)=\mathrm{clip}\;\left(\frac{{\mathrm{minfilter}}_{r_{a}}\left(I_{\min}\right)\left(x\right)-\tau_{a}}{s_{a}}\right).$$

C1 detects a dark clean background lifted toward gray, C2 detects attenuation of background texture, and C3 provides an absolute haze response independent of *B*. Their probabilistic soft-OR is

(5) $$d_{\mathrm{dark}}\left(x\right)=1-\left[1-v\left(x\right)\right]\left[1-t\left(x\right)\right]\left[1-a\left(x\right)\right].$$

For bright backgrounds, the texture branch and final background-adaptive response are

(6) $$d_{\mathrm{bright}}\left(x\right)=1\left[B_{Y}\left(x\right)>\tau_{b}\right]\mathrm{clip}\;\left(1-\frac{E_{I}\left(x\right)}{E_{B}\left(x\right)+\epsilon_{t}}\right),\;d\left(x\right)=\max\;\left(d_{\mathrm{dark}}\left(x\right),w_{b}d_{\mathrm{bright}}\left(x\right)\right).$$

The glare support is formed by thresholding near-saturated luminance, dilating, and smoothing:

(7) $$\ell \left(x\right)=G_{\sigma_{\ell }}\;\left(D_{r_{\ell }}^{\left(n_{\ell }\right)}\left(1\left[I_{Y}\left(x\right)>\tau_{\ell }\right]\right)\right).$$

With HSV saturation *S*, the soft chroma gate is

(8) $$c\left(x\right)=\mathrm{clip}\;\left(\frac{\tau_{S}-A_{r_{c}}\left(S\left(x\right)\right)}{\tau_{S}}\right),\;d_{g}\left(x\right)=G_{\sigma_{d}}\;\left(d(x)c(x)[1-\ell (x)]\right).$$

Thus, $d_{g}$ is the default continuous teacher output. The gates are not hardware stages; they are deterministic image operations, and their genuine-dust cost is reported by the gate ablations.

The optional temporal extension is evaluated separately. For a *K*-frame buffer, temporal standard deviation and Farnebäck-flow magnitude/divergence form $m_{t}=\max\left(m_{\mathrm{std}},m_{\mathrm{flow}}\right)$, and

(9) $$d_{\mathrm{temp},t}\left(x\right)=d_{g,t}\left(x\right)\left[\eta +\left(1-\eta \right)m_{t}\left(x\right)\right].$$

Because optical flow adds latency and increased clean false-positive area in the audit, this branch is excluded from the default deployment path and from the reported 610 FPS.

### 3.4. Label-Free Evaluation Data and Condition Definitions

Because no public large-scale pixel-labeled underground-dust dataset is available, we construct one synthetic validation/calibration set and one independently annotated real test set; neither is used to train the proposed teacher. The synthetic set uses atmospheric-scattering compositing, $I_{\mathrm{dusty}}=I_{\mathrm{clean}}\left(1-\alpha \right)+A\alpha$, where $\alpha \in {\left[0,1\right]}^{H\times W}$ is a fractal-noise plume opacity map and *A* is the airlight color. Twelve clean base frames are drawn only from clips assigned to the synthetic role, and 12 independently seeded plumes are generated from each base, giving 144 samples. The known compositing opacity α, or its thresholded mask for binary metrics, provides a reproducible calibration target rather than an exact real-world dust-boundary annotation. This set is used to select operating thresholds and to provide the diagnostic cue ablation in Section 4.3; it is therefore not presented as an independent held-out test benchmark. For DustVeil, every synthetic image is scored against the corresponding camera’s independently constructed background-role calibration, exactly as in real inference; the paired clean base retained by the generator is not used as the detector background. Synthetic-base clips are disjoint from all background, pseudo-label, validation, baseline-fitting, and real-test clips and sessions.

The final real test set contains 72 frames (37 with dust, 35 clean) drawn one frame per clip from 72 distinct clips in 18 global sessions, stratified by dust level across all six cameras. The frame list and its source clips were fixed before operating-threshold selection and final scoring. Two annotators—one computer-vision researcher and one mining-domain engineer—independently reviewed each target frame at 960 × 543 in a polygon annotation interface together with 1.0 s of temporal context on either side, followed a written annotation guide, and were blinded to every detector output. A plume was included when veiling or texture attenuation remained visible across at least three adjacent frames; the polygon followed the furthest consistently visible semi-transparent boundary. Isolated single-frame wisps, head-lamps, hard reflections, and solid machinery were excluded. The annotators first assigned dust/clean frame labels and then drew coarse polygons on dust frames. A third senior underground-machine-vision researcher adjudicated condition or boundary disagreements, and the adjudicated masks form the primary reference standard. Inter-annotator agreement and the sensitivity of the main comparison to each individual annotation are reported in Section 4.8. These annotations are used only for final metric computation and condition/camera breakdowns; the evaluation program does not resolve or open the real-test index, images, or annotations until every continuous-score threshold has been selected on the synthetic masks and atomically written to disk.

#### Task Scope and Reference Standard

The primary 72-frame real masks delineate visually discernible plume regions in image space. Accordingly, the reported IoU, F1, and false-positive-area metrics quantify agreement with these visual annotations only. No synchronized gravimetric or optical dust-concentration measurements, particle-size or respirable-fraction measurements, personal-exposure records, or independently recorded operational-event labels were available for the collected footage. DustVeil produces a dimensionless visual response score and a thresholded image-space mask; neither output is calibrated in units of dust mass concentration. The outputs should therefore not be interpreted as estimates of respirable-dust concentration, worker exposure, health risk, plume source, or abnormal-operation probability. DustVeil is intended to complement, rather than replace, physical dust-monitoring instruments. The separate specificity set in Section 3.7 uses condition-specific dust and water-spray masks only to quantify empirical visual selectivity; it does not convert the camera output into a physical aerosol-composition measurement.

#### Evaluation Metrics

For predicted mask *M* and visual reference *G*, intersection-over-union and F1 are

(10) $$\mathrm{IoU}=\frac{\left|M\cap G\right|}{\left|M\cup G\right|}=\frac{\mathrm{TP}}{\mathrm{TP}+\mathrm{FP}+\mathrm{FN}},\;F1=\frac{2\mathrm{TP}}{2\mathrm{TP}+\mathrm{FP}+\mathrm{FN}},$$

and clean-frame false-positive area is |M|/(HW). These are segmentation-agreement metrics, not physical concentration metrics [40,41]. IoU and F1 are averaged over the 37 positive frames; false-positive area is averaged over the 35 clean frames.

Before any frame extraction, all 493 clips in the original six-role corpus are entered exactly once in a fixed core four-column manifest (camera, clip, role, global session ID) and assigned to one of six roles: per-camera background calibration, pseudo-label training, pseudo-label validation, anomaly-baseline normal fitting, synthetic-base generation, or final real testing. The global session ID is mandatory and groups all clips from one continuous event, including synchronized recordings from different cameras. A clip or session can occur in only one role. Consequently, no final-test clip or session contributes to the background median, normal-baseline fitting, a synthetic clean base, a teacher pseudo-label, or either side of the student’s train/validation split. For descriptive auditing only, each source clip was additionally assigned one coarse visual-condition tag—clean, dust-dominant, spray-dominant, mixed, or uncertain—after the six-role allocation had been locked. These tags were not used as training labels, for role assignment, for threshold selection, or to exclude difficult clips.

Table 2 reports the realized distribution of raw clips, unique global sessions, camera coverage, coarse visual conditions, and extracted or derived units across the six roles. The role-specific clip counts sum exactly to 493.

The finalized audit contained 493 rows and 493 unique clip identifiers, with 0 missing camera fields, 0 missing role fields, 0 missing global-session fields, 0 duplicate clip rows, 0 clips assigned to more than one role, and 0 global sessions assigned to more than one role. The real-test package had 0 clip overlap and 0 session overlap with the other five roles. The complete anonymized row-level manifest, per-camera role matrix, condition summary, validator report, and SHA-256 manifest hash are supplied as Supplementary Data S1, Supplementary Tables S1 and S2, and Supplementary Audit S1. The five coarse visual-condition tags were assigned retrospectively after the six-role manifest had been locked; the predefined role-eligibility rules still required visually clean footage for background, baseline-normal, and synthetic-base roles. The added tags were used only for descriptive reporting, not as training labels, threshold labels, or grounds for post hoc exclusion. The separately held-out specificity package is maintained in its own manifest; its cross-manifest audit reports 0 overlapping clips and 0 overlapping sessions with the original corpus.

At the camera level, the protocol is intentionally not camera-disjoint. DustVeil is deployed after unlabeled, camera-specific calibration, and each of the six fixed cameras therefore contributes separate calibration and evaluation clips. This study consequently measures within-camera generalization under strict clip- and global-session-level separation, rather than zero-calibration transfer to an unseen camera. No real annotation from any camera enters calibration, training, validation or threshold selection, and the per-camera results in Section 4.8 make the camera-to-camera variation explicit. As an additional threshold-transfer diagnostic, each camera is excluded in turn from synthetic threshold selection while retaining its own unlabeled background calibration; this leave-one-camera-out analysis tests transfer of the global operating threshold across the six recorded cameras, but it does not constitute validation on an unseen mine or a camera deployed without calibration.

For each positive real frame, let *G* denote the annotated dust mask and $\rho_{G}=∥G∥/\left(HW\right)$ its area ratio. To avoid reducing plume extent to a single heavy/thin split, positive frames are summarized in five pre-specified area bins: 0–3%, 3–7%, 7–12%, 12–20%, and above 20%. We additionally report the session-bootstrap Spearman association between $\rho_{G}$ and per-frame IoU. For clean frames, the condition labels used in the later per-camera and condition analyses are assigned by simple frame-level brightness and saturation rules used only for reporting: colored machinery if $\rho_{S}>\tau_{\rho ,S}$, where $\rho_{S}=\mathrm{mean}\left(1\left[S>\tau_{S,\mathrm{cond}}\right]\right)$; head-lamp glare if $\rho_{Y}>\tau_{\rho ,Y}$, where $\rho_{Y}=\mathrm{mean}\left(1\left[I_{Y}>\tau_{Y,\mathrm{cond}}\right]\right)$; and plain clean otherwise.

### 3.5. Distillation to a Real-Time Student

The gated teacher map is binarized at pseudo-label threshold $\theta_{\mathrm{PL}}$: $Y_{T}\left(x\right)=1\left[d_{g}\left(x\right)\geq \theta_{\mathrm{PL}}\right]$. TinyU-Net minimizes Dice loss plus class-balanced binary cross-entropy and uses no human training masks. Table 3 specifies every block and tensor shape. A Conv block is two repetitions of 3 × 3 convolution, batch normalization and ReLU. Downsampling uses 2 × 2 max pooling; decoding uses bilinear upsampling followed by concatenation with the matching encoder feature.

Pseudo-label frames are sampled at indices 60, 125, and 190, producing 270 training frames from 90 clips/18 sessions and 72 validation frames from 24 clips/6 sessions. Complete sessions remain in one split. Training uses the augmentation and equal-budget settings given below.

### 3.6. Implementation Details

The teacher runs at 960 × 543 with the constants reported in the released configuration. TinyU-Net and the three architecture references run at 512 × 288. All decoded OpenCV arrays are converted from BGR to RGB before tensor construction. TinyU-Net, trained from scratch, scales RGB values to [0, 1]. Pretrained LR-ASPP-MobileNetV3 and DeepLabV3-ResNet50 additionally use ImageNet mean [0.485, 0.456, 0.406] and standard deviation [0.229, 0.224, 0.225]; SegFormer-B0 uses the corresponding model-processor RGB resize and normalization. The corrected comparison therefore does not feed raw BGR [0, 1] tensors to pretrained backbones.

For distillation, $\theta_{\mathrm{PL}}=0.40$, base width is 24, and training uses 60 epochs, effective batch size 8, Adam, identical pseudo-label splits, loss, augmentation, learning-rate grid, and seeds 11, 23 and 37 for all four architectures. Horizontal flip, moderate brightness/contrast and gamma perturbation, Gaussian noise and blur are used; vertical flip, large rotation, random crop and hue/saturation jitter are excluded to preserve fixed-camera geometry and color evidence.

The label-free baselines are evaluated with a common fixed calibration rule. Per-image min–max normalization is not used. For each method and camera, the 1st and 99th percentiles are estimated once from the 50 dedicated baseline-normal frames, stored, and then applied unchanged to synthetic, real-test, and spray-specificity maps: $s^{\prime }=\mathrm{clip}\left(\left(s-q_{1}\right)/\left(q_{99}-q_{1}\right)\right)$. DCP and wavelet responses already have bounded definitions and are clipped to [0, 1]. A single global binary threshold per method is selected only on synthetic masks. This preserves absolute between-image score variation and prevents clean-test frames from defining their own dynamic range.

PaDiM is implemented through a documented reference adaptation; PatchCore and SimpleNet use the authors’ public architectures and scoring pipelines with only dataset and input adapters; EfficientAD uses the published PDN/student–teacher/autoencoder design through a reference implementation rather than the earlier simplified feature-reconstruction surrogate. Version pins, preprocessing, normal-fit data, score extraction, and calibration are recorded in Supplementary Table S8 and the code README. Grounded-SAM2 uses the fixed prompt coal dust. airborne dust cloud., box threshold 0.20 and text threshold 0.18, with no additional morphology; the complete protocol is reported in Table 4.

The reproducibility package now exposes the central reported procedures rather than only the main inference path. It includes scripts for baseline score calibration, student RGB/model preprocessing, gate ablation, temporal replay, and network/end-to-end timing; frozen JSON configurations; expected CSV schemas; and automated smoke tests. The gate and temporal scripts emit the corresponding fixed-schema CSV outputs, and the benchmark script separates network-only, decoded-frame, single-stream, and six-stream scopes. Raw operational video remains access-controlled, but the commands, manifests, derived audit tables, and synthetic smoke test are supplied.

### 3.7. Dust-Versus-Water-Spray Specificity Evaluation

To test whether the detector responds to the target dust-plume appearance rather than to any diffuse aerosol, we created a separate held-out specificity package comprising 96 frames from 24 global recording sessions and 6 cameras. Its source clips and sessions were not used for background construction, pseudo-label generation, student training or validation, anomaly-baseline fitting, synthetic threshold calibration, or the primary 72-frame real test. The frame list was fixed before any method output on the specificity package was inspected, and selected frames from the same clip were separated by at least 2.0 s to reduce near-duplicate sampling.

Two annotators—one computer-vision researcher and one mining-domain engineer—independently reviewed 2.0 s of temporal context before and after each candidate frame in randomized order, without access to any model prediction or pre-existing mask, and assigned one of four condition labels: dust-only, spray-only, mixed dust and spray, or uncertain. Dust-only and spray-only labels required clear temporal context: cutting-related airborne dust without visible spray, or active water spray without visible cutting dust, respectively. The annotators independently drew a dust polygon on each dust-only frame and a water-aerosol polygon on each spray-only frame. A third senior researcher with underground-machine-vision experience resolved label or mask disagreements before scoring. Condition decisions used the visible spray-nozzle state, shearer cutting activity and full temporal context rather than the isolated RGB frame. Mixed frames were retained to document the confounded condition but were excluded from the primary pixel-level specificity metrics because source-specific boundaries were not unambiguous in a single RGB view; uncertain frames were excluded from all method comparisons. The following unnumbered protocol table reports the resulting set composition, see Table 5.

All methods use exactly the same preprocessing, post-processing, and operating thresholds as in the primary evaluation. For every continuous-score method, the threshold stored by the synthetic-only calibration procedure is loaded before the specificity package can be opened; Grounded-SAM2 retains its fixed box and text thresholds. No model parameter, gate, prompt, threshold, or post-processing rule is selected using the specificity labels.

On dust-only frames, performance is measured against the dust masks using IoU, per-frame F1, and recall. On the $N_{s}$ spray-only frames, any predicted dust region is a false response. For method *m*, with frozen binary prediction ${\hat{M}}_{i}^{\left(m\right)}$ and annotated water-spray mask $S_{i}$, we report the mean predicted area

$${\mathrm{MPA}}_{\mathrm{spray}}^{\left(m\right)}=\frac{100}{N_{s}}\sum_{i=1}^{N_{s}}\frac{|{\hat{M}}_{i}^{\left(m\right)}|}{HW},$$

the mean spray-mask response ratio

$${\mathrm{SMR}}^{\left(m\right)}=\frac{100}{N_{s}}\sum_{i=1}^{N_{s}}\frac{|{\hat{M}}_{i}^{\left(m\right)}\cap S_{i}|}{|S_{i}|+\epsilon },$$

and frame-FP@5%, the percentage of spray-only frames for which the predicted dust area exceeds 5% of the image. Lower values are better for all three spray-only measures. This paired reporting measures dust retention and water-aerosol response under one frozen operating point. Uncertainty for the dust-only and spray-only outcomes is obtained with 2000 paired session-clustered percentile resamples using random seed 20,260,812, so temporally related frames from one recording session are never treated as independent observations.

### 3.8. Quantitative Robustness and Deployment Audits

#### Background Quality, Temporal Drift, and Sensitivity

Within each camera, cross-session background consistency is measured by leaving out one background-role global session, constructing $B_{c}^{\left(-s\right)}$ from all remaining sessions, and comparing it with the temporal median $R_{c,s}$ of the held-out session. Absolute background quality is additionally checked against 60 manually verified clean frames from 12 sessions (10 frames per camera), selected without viewing detector outputs. We report luminance normalized mean absolute error (NMAE), SSIM, correlation of local gradient-energy maps, and the image fraction $A_{20}$ whose absolute luminance difference exceeds 20 gray levels. Temporal drift is measured by constructing separate backgrounds from the earliest and latest halves of each camera’s chronologically ordered calibration sessions; the median early–late separation is 14.0 [7.0, 21.0] days.

Downstream sensitivity is evaluated on the unchanged 72-frame real set using six background constructions: the nominal full temporal median, early-half median, late-half median, a 25% session-sparse median, a temporal mean, and a single-frame reference. Sparse and single-frame variants are repeated with the five fixed random seeds 11, 23, 37, 41 and 53. All cue constants, gates and synthetic-selected operating thresholds remain frozen. On the 342 pseudo-label frames, we additionally report Dice agreement and symmetric changed-pixel area relative to the nominal teacher labels. To propagate this sensitivity through distillation, TinyU-Net is also retrained with the early-half-background pseudo-labels using the same three seeds and optimization budget as the nominal student.

#### Session-Clustered Uncertainty and Annotation Sensitivity

The 72 frames originate from 18 global sessions (10 dust-containing and 8 clean sessions). Confidence intervals are therefore obtained with 2000 paired, condition-stratified cluster-bootstrap resamples using random seed 20,260,812: dust-containing and clean sessions are resampled separately with replacement, global sessions rather than individual frames are the sampling units, and all frames from an included session enter every method’s metric calculation. Paired differences between DustVeil and DCP are computed within each identical bootstrap sample. The primary metrics are also recomputed against each annotator’s pre-adjudication masks to quantify sensitivity to the coarse boundary reference.

#### Operating and Pseudo-Label Threshold Sensitivity

The final teacher threshold $\tau_{m}$ is swept from 0.25 to 0.50 while all cue and gate constants remain fixed. The nominal value is the synthetic-selected threshold and the real masks are used only to report the post-hoc sensitivity curve. For $\theta_{\mathrm{PL}}\in \left\{0.30,0.35,0.40,0.45,0.50\right\}$, pseudo-labels are regenerated and TinyU-Net is retrained with the same 270/72-frame session-disjoint split, augmentation policy, optimizer budget, and seeds 11, 23 and 37. We report pseudo-label foreground area, pseudo-validation Dice, real IoU/F1, and clean false-positive area.

#### Frame-Area Exceedance and Continuous Plume-Extent Analysis

The clean-frame exceedance function is evaluated at predicted-area thresholds 0.5, 1, 2, 3, 5, 7.5, 10, 15 and 20%. The 1%, 5%, and 10% values retained in the main comparison correspond to approximately 5,213, 26,064, and 52,128 pixels at 960 × 543 and were fixed as interpretable small, conspicuous, and large response anchors. The full exceedance curve and its normalized area are reported in Supplementary Table S15. Positive-frame IoU is reported in the five pre-specified $\rho_{G}$ bins, and its association with plume area is quantified with a session-bootstrap Spearman coefficient.

#### Synthetic-to-Real Calibration and Camera Scope

After the primary threshold and real-test results have been locked, a post hoc diagnostic compares the synthetic-selected threshold with the real-test oracle threshold; the oracle is reported only to quantify transfer regret and is never used to update a headline result. The synthetic generator is also perturbed in opacity, airlight color, plume morphology and illumination interaction. The opacity field is multiplied by 0.8 or 1.2 and clipped to [0, 1]; nominal gray airlight A=(210,210,210) is replaced by warm A=(225,205,185) or cool A=(185,205,225) airlight; the plume field is spatially scaled by 0.75 or 1.25; lamp-adjacent placement enforces 30–50% plume-mask overlap with the precomputed glare support; low-illumination placement restricts the plume centroid to the lowest luminance quartile; and spatially varying airlight applies a linear 0.85–1.15 intensity gradient across the plume. A fresh threshold is selected on each perturbed synthetic set and then applied unchanged to the real set. For leave-one-camera-out threshold transfer, synthetic samples from one camera are excluded during threshold selection, while that camera retains its own unlabeled background calibration and remains held out from all threshold fitting.

#### Gate, Temporal, and Runtime Audits

The glare and chroma gates are ablated on both dust and clean real frames. Every gate variant uses the same nominal frozen final threshold; only the gate state is changed, and no variant-specific threshold retuning is permitted. Dust-over-lamp and dust-over-coloured-machinery subsets are defined when at least 5% of the annotated dust mask intersects the corresponding dilated lamp or high-saturation support. The exploratory temporal stage is evaluated on 18 session-disjoint three-second windows (1350 frames) using flow-warped temporal IoU, changed-mask flicker area, and added latency. Labeled IoU and clean false-positive area are computed on the unchanged 72 labeled target frames after replaying the six preceding frames; the 1,350 unlabeled window frames are used only for temporal consistency and flicker. No event-level metric is claimed because operational-event labels are unavailable. Runtime is measured in FP32 after 200 warm-up iterations over 5000 frames, with CUDA synchronization, on an NVIDIA RTX 4070 Ti and Intel Core i9-13900K. H.264 software decoding is performed through FFmpeg-backed OpenCV workers with one worker and a bounded queue of 2 frames per stream. A six-stream scheduling cycle processes one frame from each stream; cycle latency and aggregate throughput are therefore reported separately. Network-only, decoded-frame pipeline, full decode-to-mask single-stream, and six-stream concurrent scopes are reported separately.

## 4. Results

### 4.1. Manifest Realization and Evaluation Protocol

The realized corpus allocation is reported in Table 2. The manifest contains 493 unique clips from 107 global sessions across six cameras. The validator found zero duplicated clip assignments, zero cross-role clip overlap, zero cross-role session overlap, and no real-test clip or session used by background construction, pseudo-label generation, student validation, anomaly-baseline fitting, or synthetic threshold calibration. These observed audit results, rather than the role rules alone, support the leakage-control claim.

Performance is evaluated with the definitions in Equation (10). For every continuous method, one global threshold $\tau_{m}$ is selected by maximizing mean IoU on the 144 synthetic masks over {0.10,0.125,…,0.80}. The calibration record is written to frozen_thresholds.csv, while the manifest hash, score-calibration percentiles, software versions, and grid are written to threshold_audit.json; both files are stored in outputs/calibration/. Only after these records exist does the evaluation command resolve the real-test index. The same frozen mapping and threshold are used for every real and spray-specificity frame.

### 4.2. Comparison with Label-Free Methods

Table 6 reports the corrected comparison. The previous released baseline path normalized each score map independently, which removed absolute between-frame scale and inflated false-positive area. The revised implementation fits one score calibration per method and camera only on the baseline-normal role, freezes it, and applies it to every subsequent map. PatchCore, SimpleNet, and EfficientAD are also run through reference implementations rather than simplified surrogates. Under this protocol, DustVeil retains the lowest clean false-positive area (3.7%), while DCP remains statistically comparable in IoU/F1. The anomaly baselines improve substantially relative to the per-image-normalized audit, but still produce 13.9–24.9% clean false-positive area. Grounded-SAM2 remains the largest false-response method because its binary masks cannot be recalibrated by a pixel-score transform.

**Figure 2:**
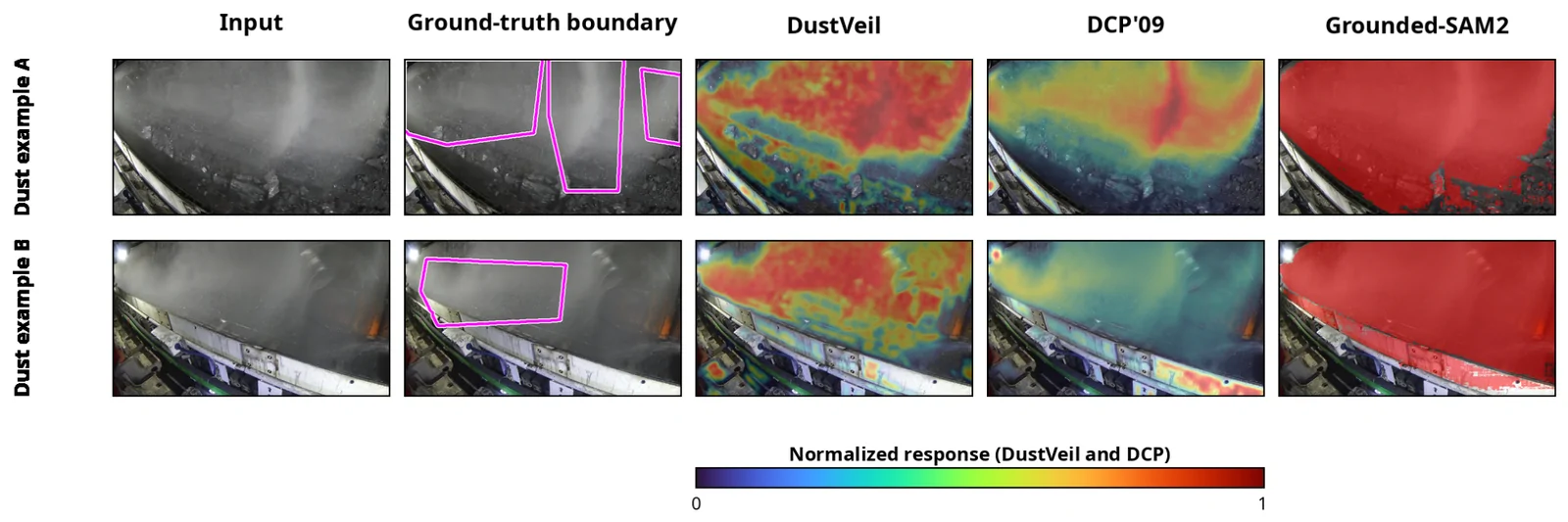
Enlarged dust-containing examples. Columns show input, adjudicated ground-truth boundary, DustVeil, DCP, and Grounded-SAM2. The magenta contour with a white halo denotes the adjudicated ground-truth plume boundary. DustVeil and DCP are displayed on a common normalized response scale of [0, 1]; Grounded-SAM2 is a binary overlay. This dust-positive panel is retained as a separate figure from the clean-glare panel to preserve readability at manuscript scale.

**Figure 3:**
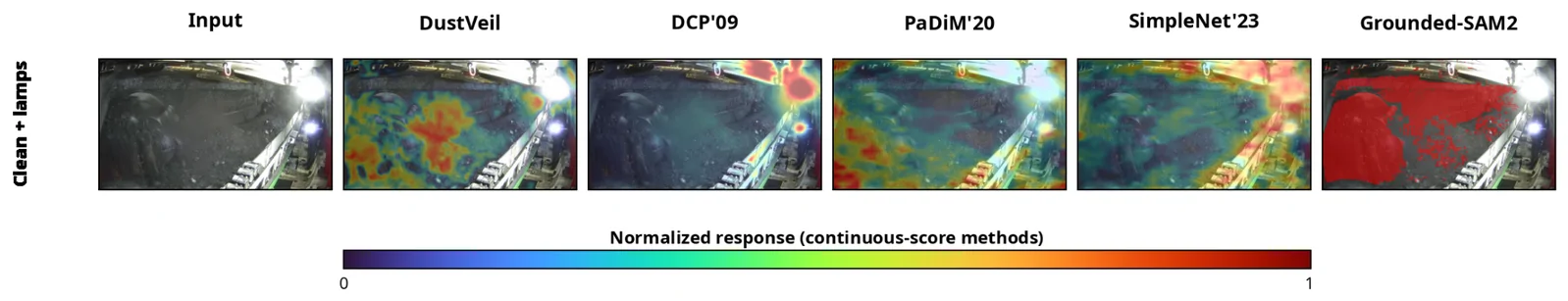
Enlarged clean-frame comparison under head-lamp glare. Continuous score maps use the same normalized [0, 1] color range. The broad non-dust responses visually correspond to the clean-frame exceedance statistics. This clean-glare panel is retained separately from the dust-positive panel to preserve detail.

Supplementary Table S20 reports the paired implementation audit: changing only per-image normalization to fixed baseline-normal calibration reduces the clean false-positive area of Wavelet, PaDiM, PatchCore, SimpleNet, and EfficientAD from 61.2, 90.2, 96.9, 99.8, and 78.0% to 22.6, 24.9, 19.7, 16.8, and 13.9%, respectively. Figure 2 and Figure 3 use the same fixed display mapping as the quantitative evaluation.

### 4.3. Complete Cue-Combination Ablation

Table 7 forms the first part of the complete component ablation. For each ungated cue set, its threshold is selected on the synthetic calibration masks and then frozen for the real test. Gate effects are reported subsequently without variant-specific threshold retuning. Among the ungated cue sets, C1 + C2 + C3 gives the highest real-test IoU (0.389), while the full three-cue response reaches recall 0.695 before suppression. The coordinated cue and gate ablations therefore show that the final operating point is not obtained by an unreported sequence of tuning decisions: pairwise cue complementarity is measured first, and the two gates then trade recall for a substantially lower clean-frame false-positive area.

### 4.4. Operating- and Pseudo-Label-Threshold Sensitivity

The teacher operating threshold has a broad performance region: thresholds 0.325–0.425 remain within 0.007 IoU of the best observed value, while the nominal synthetic-selected value 0.375 yields the maximum IoU/FP compromise. The pseudo-label threshold also shows a stable central region (0.35–0.45); very low values increase foreground noise and false positives, whereas very high values remove faint plume supervision. The complete sweeps are supplied in Supplementary Table S14; Table 8 reports the central operating region.

### 4.5. False-Alarm Gate Trade-Offs and Exploratory Temporal Extension

Table 9 reports the four gate configurations under one frozen threshold. The full gate ablation shows an explicit false-response/recall trade-off: both gates reduce clean false-positive area from 14.7% to 3.7%, while overall recall changes from 0.695 to 0.566. The default is therefore a conservative low-false-response operating point rather than a universally superior segmentation configuration. The largest recall costs occur on the 9 dust frames overlapping lamp support and the 8 frames overlapping colored machinery, so the ablation reports the practical cost of suppression rather than only its benefit on clean frames. Table 10 then quantifies the genuine-dust pixels removed by the chroma gate. On the 8 colour-overlap dust frames, the chroma gate removes a mean 12.4% of annotated dust pixels (median 9.8%); 5/8 frames lose more than 10% and 2/8 lose more than 20%. The worst observed frame loses 31.6%, quantifying the cost behind the lower colour-overlap recall in the gate ablation.

Table 11 reports the exploratory temporal comparison. The exploratory temporal extension improves flow-warped consistency from 0.741 to 0.812 and reduces flicker from 4.3% to 2.9%, but increases clean false-positive area from 3.7% to 4.6% and adds 7.4 ms per frame; it is therefore excluded from the default deployment path. The labeled detection metrics are computed on the unchanged 72-frame reference set after buffer replay, whereas the temporal-consistency and flicker metrics use only the 1350 unlabeled frames from 18 held-out video windows.

### 4.6. Equal-Budget Student Comparison and Deployment Timing

After explicit BGR-to-RGB conversion and architecture-appropriate input normalization, LR-ASPP, SegFormer-B0 and DeepLabV3 obtain mean IoUs of 0.432, 0.414 and 0.405, respectively. TinyU-Net remains the smallest and fastest student, whereas LR-ASPP provides the strongest IoU/F1 under the equal optimization budget. The student uses moderate geometry-preserving and photometric augmentation. Relative to no augmentation, the standard policy changes pseudo-validation Dice from 0.817 to 0.842, real IoU from 0.386 to 0.398, and clean FP from 5.7% to 4.9%. Table 12 reports the harmonized comparison.

Table 13 separates network-only timing from decoded-frame, single-stream, and six-stream deployment scopes. The runtime audit separates the 610 FPS forward pass from the 147 FPS single-stream decode-to-mask path. Six concurrent streams achieve 233 FPS aggregate at the median and 204 FPS at p95 cycle latency, exceeding the 150 FPS aggregate input rate of six 25-FPS streams on the measured hardware. One six-stream scheduling cycle processes six frames—one from each stream—so aggregate throughput is computed as six divided by cycle latency. The per-stream median throughput is 38.8 FPS, the measured drop rate is 0.0%, and the queue depth is 2 frames per stream. The teacher and exploratory temporal branch are excluded because the teacher is offline and the temporal branch is not the default deployment configuration.

### 4.7. Qualitative Results

**Figure 4:**
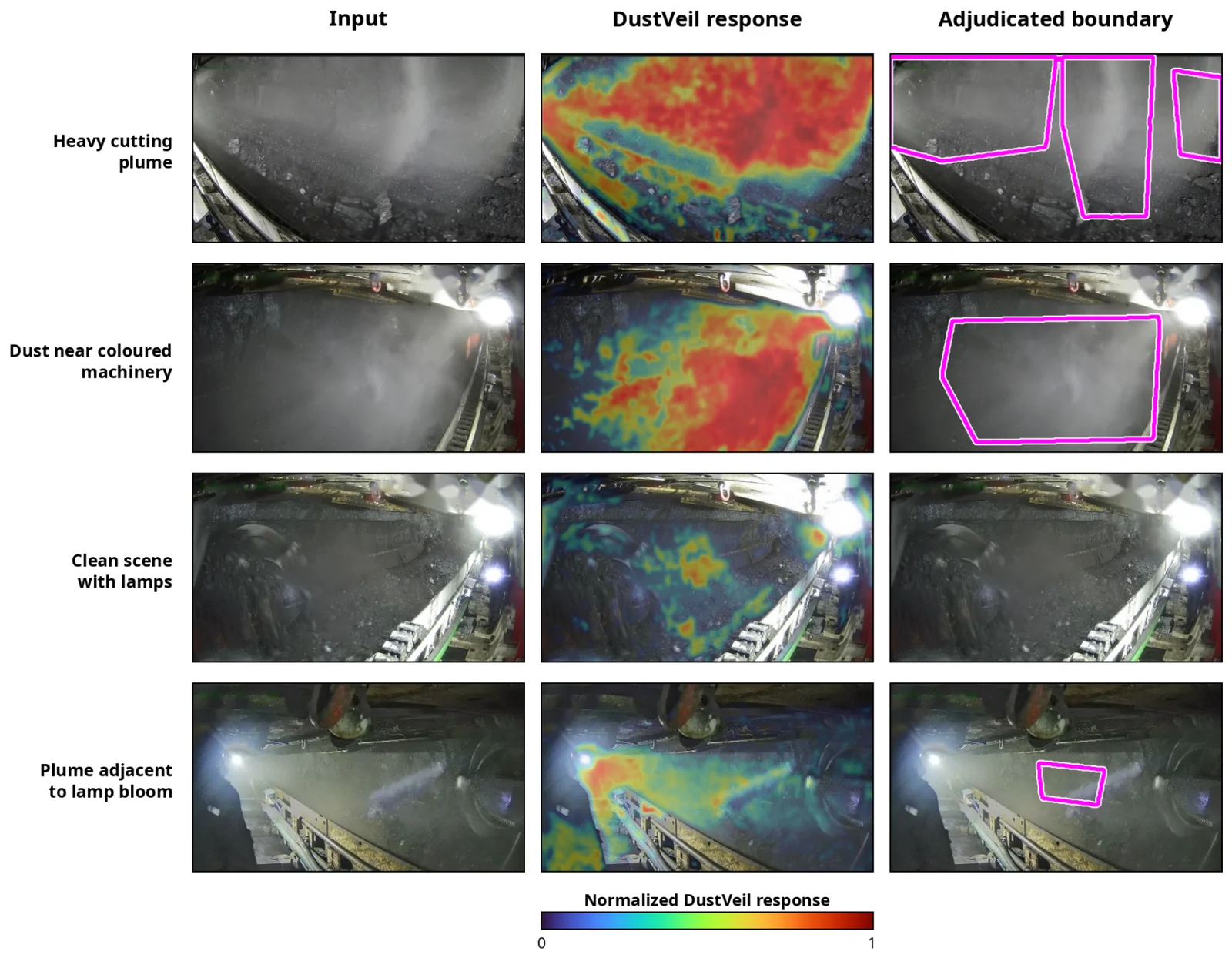
Representative DustVeil outputs. Rows show a heavy cutting plume, dust near colored machinery, a clean scene with lamps, and an annotated plume adjacent to strong lamp bloom. Columns show the input, normalized DustVeil response, and adjudicated ground-truth boundary. The magenta contour with a white halo denotes the adjudicated plume boundary; no contour is shown for clean rows. The separately defined spray-only condition is evaluated quantitatively in Section 4.9.

Figure 4 shows larger DustVeil outputs for plume, lamp, colored-machinery, and clean conditions. A common [0, 1] color scale is used, and the adjudicated reference is shown as a magenta boundary with a white halo for visibility.

### 4.8. Robustness, Annotation, and Calibration Audits

#### Annotation Reliability

Before adjudication, the two annotators achieved Cohen’s κ=0.91 for dust/clean frame labels. On the 37 positive frames, the mean pairwise mask IoU was 0.79 [0.72, 0.85], Dice was 0.88 [0.84, 0.92], and the dust-area intraclass correlation was 0.94 [0.89, 0.97] (Table 14). Adjudication was required for 14/72 (19.4%) frames. DustVeil’s IoU was 0.354, 0.361 and 0.366 against annotator A, annotator B, and the adjudicated mask, respectively; DCP remained lower at 0.347, 0.353, and 0.358. The comparative interpretation is therefore stable to the individual coarse boundary reference.

#### Background Quality and Drift

Against the manually verified clean-reference subset, the temporal-median backgrounds obtain luminance NMAE 0.027 ± 0.009, SSIM 0.941 ± 0.018, gradient-energy correlation 0.901 ± 0.025, and changed area 5.2 ± 1.8%. The leave-one-session-out analysis gives NMAE 0.031 ± 0.010 and SSIM 0.928 ± 0.022, while the early-to-late comparison over a median gap of 14.0 [7.0, 21.0] days gives NMAE 0.041 ± 0.012 and SSIM 0.906 ± 0.026 (Table 15).

Table 16 reports the effect of alternative background constructions. Chronologically separated median backgrounds show bounded sensitivity: the early- and late-half IoU reductions are 0.015 and 0.007. A temporal mean and a single-frame reference increase clean false-positive area to 5.6% and 8.9 ± 1.4%, respectively, while pseudo-label agreement decreases in the same order. Retraining TinyU-Net from the early-half-background pseudo-labels produces IoU 0.388 ± 0.007 and clean FP 5.4 ± 0.4%, compared with 0.398 ± 0.006 and 4.9 ± 0.3% under the nominal labels, showing that the observed teacher-label sensitivity causes a limited but measurable downstream student change.

#### Session-Clustered Uncertainty

The 72 frames are grouped into 10 dust-containing and 8 clean global sessions. The paired session-clustered analysis does not establish an IoU advantage over DCP (difference 0.008 [−0.021, 0.038]), but it supports a lower false-positive area (difference −11.1 [−14.4, −8.1] percentage points). The same session draws are applied to every method, preserving paired comparisons while avoiding invalid resamples that contain only one condition. Table 17 reports the resulting intervals.

#### Synthetic-to-Real Threshold Transfer

The nominal synthetic set selects τ=0.375, yielding synthetic IoU 0.603 and real IoU 0.366. The post hoc real-oracle threshold is 0.400 with IoU 0.378, the synthetic–real threshold-curve correlation is 0.84, and the near-optimal real plateau is [0.350, 0.425]. The synthetic-selected threshold lies inside the reported real-performance plateau, with post-hoc IoU regret 0.012. Across opacity, airlight, morphology and illumination-interaction perturbations, the real IoU spread is 0.014; this supports the frozen synthetic-only operating point within the recorded domain, but not physical realism or transfer to a different mine. Table 18 reports the generator-perturbation audit.

#### Leave-One-Camera-Out Threshold Diagnostic

Excluding each camera from synthetic threshold selection produces the results in Table 19. Aggregate IoU is 0.356 versus 0.366 in the nominal all-camera calibration, and aggregate FP is 3.9% versus 3.7%. This indicates that the global threshold is not driven by a single camera among the six tested streams. It does not validate zero-calibration transfer, a new camera geometry, or a new mine.

Per-camera. Table 20 shows that IoU and F1 vary considerably across the six cameras (IoU 0.15 to 0.72), reflecting differences in viewpoint, dust density, and lighting, whereas the false-positive rate remains low (1.5 to 5.9%) for all of them.

Plume extent. Table 21 reports IoU, F1, and recall across five annotated dust-area bins instead of a binary heavy/thin split. The dense clean-frame exceedance curve confirms that the three reported anchors are not selectively chosen: the normalized area-under-exceedance curve over 0.5–20% is 16.5 for DustVeil and 78.6 for DCP. On positive frames, IoU increases monotonically across the five pre-specified dust-area bins, with a session-bootstrap Spearman association of 0.68 [0.47, 0.81].

Frame-level false-positive responses. The 1%, 5%, and 10% anchors correspond to approximately 5,213, 26,064, and 52,128 pixels at 960 × 543. The dense curve in Supplementary Table S15 avoids relying only on these anchors. Under fixed score calibration, DustVeil exceeds 5% predicted area on 26% of clean frames; the corresponding rates are 97% for DCP, 71% for EfficientAD, and 100% for Grounded-SAM2. These are visual false-response rates, not operational alarms. Table 22 reports the three pre-specified exceedance anchors.

### 4.9. Dust-Versus-Water-Spray Specificity

The held-out specificity set comprised 96 frames from 24 independent recording sessions and 6 cameras; its composition is summarized in the unnumbered protocol table in Section 3.7. Before adjudication, the two annotators achieved a condition-label agreement of Cohen’s κ=0.88; their mean pairwise mask IoU was 0.79 for dust-only frames and 0.76 for spray-only frames. The 18 mixed frames and 6 uncertain frames were excluded from the primary quantitative comparison according to the pre-specified protocol.

Table 23 reports both target-dust retention and spray-only false response under the unchanged frozen operating point. Under the unchanged frozen operating point, DustVeil retains dust-only IoU/recall of 0.371/0.579 and produces spray-only mean area/spray-mask response of 4.1%/12.6%, below DCP’s corresponding 15.9%/44.8%. These results demonstrate empirical visual selectivity within the recorded conditions, not physical aerosol-composition identification. Session-clustered 95% intervals are [0.305, 0.438] for DustVeil dust-only IoU, [2.7, 5.9] for its spray-only predicted area, and [8.1, 18.4] for its spray-mask response; the corresponding DCP intervals are [0.294, 0.431], [12.0, 20.5], and [36.3, 53.4].

## 5. Discussion

The revised analysis positions DustVeil as a low-false-response visual localizer rather than a physical dust meter. The paired session-clustered comparison does not establish an IoU advantage over DCP (difference 0.008 [−0.021, 0.038]), but supports a lower clean false-positive area (difference −11.1 [−14.4, −8.1] percentage points). The full cue and gate tables show why: cue fusion increases recall, while glare and chroma suppression move the selected operating point toward fewer conspicuous clean responses at a measurable cost in recall.

Reviewer-driven implementation audits materially changed the comparison. Per-image min–max normalization was removed because it assigns every image its own dynamic range and makes absolute false-response rates incomparable. Fixed baseline-normal calibration lowered the anomaly-baseline false-positive areas, but DustVeil remained lowest. Likewise, explicit RGB conversion and model-specific normalization improved the pretrained student backbones. These corrections are recorded in Supplementary Tables S20 and S21 and in executable audit scripts, so the conclusions no longer depend on the earlier preprocessing paths.

The annotation, background, threshold, and spray audits define the evidential boundary. Coarse plume masks are reproducible enough for comparative image-space evaluation, but semi-transparent boundaries remain subjective. Early/late and sparse-background experiments show when camera-specific recalibration is needed. The synthetic threshold lies in a broad real-performance region, but this does not prove physical realism. The water-spray experiment measures visual selectivity within the recorded conditions and does not identify aerosol composition.

The optional temporal branch improves flow-warped consistency but adds 7.4 ms/frame and increases clean false-positive area, so it is excluded from the default deployment path and from the 610 FPS claim. The default student benchmark includes correct model preprocessing, and the timing package separates network-only, decoded-frame, single-stream, and six-stream measurements.

Limitations remain substantial. The data come from one site and six fixed, individually calibrated visible-light cameras. No synchronized concentration, particle-size, respirable-exposure, or operational-event reference was available. Raw footage is access-controlled. DustVeil therefore does not support safe/tolerable/dangerous classification, certified alarm decisions, unseen-camera deployment without calibration, or direct replacement of physical dust instruments. Future work should pair the visual output with calibrated aerosol instruments and test new sites, camera geometries, and longer-term scene drift.

## 6. Conclusions

DustVeil integrates three established optical cues, underground-specific false-response gates, leakage-controlled calibration, and teacher-to-student distillation into a reproducible visual plume-localization pipeline. On the session-separated test set, it attains IoU/F1 of 0.366/0.500 and the lowest clean false-positive area (3.7%) after fair fixed score calibration of all continuous baselines. TinyU-Net provides 610 FPS network-only inference and 233 FPS aggregate in the measured six-stream pipeline; optical flow is reported separately. DustVeil is therefore a reproducible research prototype for visual plume localization on six fixed, individually calibrated visible-light camera streams, not a certified physical dust meter or a directly deployable occupational-safety decision system.

## Figures and Tables

**Table 1:** Returned outputs and their interpretation. None is a calibrated physical dust measurement.

Output	Shape/Range	Meaning	Not Supported
Visual response $D_{t}$	H×W, [0, 1]	Dimensionless evidence that the pixel resembles the target visual plume condition	No mg/m^3^, particle size or respirable fraction
Binary mask $M_{t}$	H×W, {0, 1}	Pixels above the frozen operating threshold	No source identity or aerosol composition guarantee
Area ratio $\rho_{t}$	scalar, [0, 1]	$|M_{t}|/\left(HW\right)$, the fraction of image pixels flagged	No safe, tolerable or dangerous category; no exposure decision

**Table 2:** Realized allocation of the original 493-clip corpus across the six mutually exclusive roles. The condition column reports clip-level counts as clean/dust-dominant/spray-dominant/mixed/uncertain; these descriptive tags do not enter model fitting or threshold selection. The aggregate total row is shown in bold.

Manifest Role	Clips	Sessions	Cameras	Condition Counts C/D/S/M/U	Extracted or Derived Units
background	145	29	6/6	145/0/0/0/0	145 background frames
pseudo_train	90	18	6/6	20/45/15/8/2	270 pseudo-label frames
pseudo_val	24	6	6/6	5/12/4/2/1	72 pseudo-label frames
baseline_normal	150	30	6/6	150/0/0/0/0	300 normal frames
synthetic	12	6	6/6	12/0/0/0/0	12 bases; 144 composites
real_test	72	18	6/6	35/37/0/0/0	72 annotated frames
**Total**	**493**	**107**	**6**	**367/94/19/10/3**	–

**Table 3:** TinyU-Net block structure for one input sample. The batch dimension is omitted.

Block	Operation	Output Shape	Connection
Input	RGB normalization	3 × 288 × 512	decoded BGR converted to RGB
E1	Conv block, 24 channels	24 × 288 × 512	skip to D1
E2	max-pool + Conv block, 48 channels	48 × 144 × 256	skip to D2
E3	max-pool + Conv block, 96 channels	96 × 72 × 128	skip to D3
Bottleneck	max-pool + Conv block, 96 channels	96 × 36 × 64	decoder input
D3	bilinear upsample + concat(E3) + Conv block	48 × 72 × 128	to D2
D2	bilinear upsample + concat(E2) + Conv block	24 × 144 × 256	to D1
D1	bilinear upsample + concat(E1) + Conv block	24 × 288 × 512	to output
Output	1 × 1 convolution + sigmoid	1 × 288 × 512	resized to 960 × 543 for reporting

**Table 4:** Implementation, preprocessing, and fixed score calibration of the label-free reference pipelines.

Method/Status	Fit/Input	Score Handling	Frozen Evaluation Rule
DCP/equation implementation	960 × 543 RGB; no fitting	bounded map clipped to [0, 1]	one synthetic-only threshold
Wavelet/reference implementation	960 × 543 luminance; no fitting	bounded energy map clipped to [0, 1]	one synthetic-only threshold
PaDiM/reference adaptation	WRN-50; 300 normal frames; RGB/ImageNet normalization	fixed camera/method $q_{1}$–$q_{99}$ calibration	one synthetic-only threshold
PatchCore/author-reference adapter	WRN-50; 4000-patch coreset; RGB/ImageNet normalization	fixed camera/method $q_{1}$–$q_{99}$ calibration	one synthetic-only threshold
SimpleNet/author-reference adapter	published projection/discriminator; RGB/ImageNet normalization	fixed camera/method $q_{1}$–$q_{99}$ calibration	one synthetic-only threshold
EfficientAD/reference implementation	published PDN, student and autoencoder; RGB/model normalization	fixed camera/method $q_{1}$–$q_{99}$ calibration	one synthetic-only threshold
Grounded-SAM2/pretrained models	original aspect ratio; fixed text prompt	union of returned binary masks	fixed 0.20/0.18 box/text thresholds

**Table 5:** Composition and quantitative use of the held-out dust-versus-water-spray specificity set. Counts are reported by frame, global recording session, and camera.

Condition	Frames	Sessions	Cameras	Reference Annotation	Use in Evaluation
dust-only	36	8	6	Dust polygon	Dust IoU/F1/recall
spray-only	36	8	6	Water-aerosol polygon	Spray area/SMR/frame-FP@5%
mixed	18	6	5	Condition label	Descriptive only
uncertain	6	2	2	Condition label	Excluded

**Table 6:** Corrected comparison on the 72-frame real set. Continuous baseline scores use fixed baseline-normal calibration, never per-image min–max normalization; thresholds are selected only on synthetic masks. Up arrows indicate that higher values are better, whereas the down arrow indicates that lower values are better. The best value in each metric column is shown in bold.

Method	Implementation	Dust IoU ↑	F1 ↑	Clean FP% ↓
Ours	this work	**0.366**	**0.500**	**3.7**
DCP	equation implementation	0.358	0.490	14.8
Grounded-SAM2	pretrained pipeline	0.280	0.400	59.9
Wavelet	reference implementation	0.274	0.410	22.6
PaDiM	reference adaptation	0.247	0.365	24.9
PatchCore	author-reference adapter	0.263	0.389	19.7
SimpleNet	author-reference adapter	0.281	0.406	16.8
EfficientAD	reference implementation	0.294	0.428	13.9

**Table 7:** Real-test ablation of all single and pairwise cue combinations before glare/chroma suppression. Each cue set receives one synthetic-only selected threshold, which is then applied unchanged to the real test. Higher is better for IoU/F1/recall; lower is better for clean FP. The complete three-cue configuration without gates is shown in bold.

Cue Configuration	Selected τ	IoU	F1	Recall	Clean FP%
C1 veiling	0.325	0.274	0.392	0.515	11.8
C2 texture decay	0.350	0.301	0.421	0.548	17.6
C3 absolute dark channel	0.400	0.358	0.490	0.638	14.8
C1 + C2	0.350	0.329	0.454	0.598	15.9
C1 + C3	0.375	0.376	0.516	0.674	17.2
C2 + C3	0.375	0.369	0.508	0.661	18.5
**C1 + C2 + C3 (no gates)**	**0.375**	**0.389**	**0.521**	**0.695**	**14.7**

**Table 8:** Sensitivity to the teacher operating threshold $\tau_{m}$ and pseudo-label threshold $\theta_{\mathrm{PL}}$. Each $\theta_{\mathrm{PL}}$ row retrains TinyU-Net with the same three seeds and optimization budget. The nominal values are bold.

Parameter	Value	Pseudo-FG%	Pseudo-val Dice	Real IoU	Real F1	Clean FP%
$\tau_{m}$	0.325	–	–	0.360	0.493	4.7
$\tau_{m}$	0.350	–	–	0.364	0.498	4.1
$\tau_{m}$	**0.375**	–	–	**0.366**	**0.500**	**3.7**
$\tau_{m}$	0.400	–	–	0.364	0.497	3.2
$\tau_{m}$	0.425	–	–	0.359	0.491	2.8
$\theta_{\mathrm{PL}}$	0.35	10.9	0.829	0.393	0.529	5.8
$\theta_{\mathrm{PL}}$	**0.40**	**8.6**	**0.842**	**0.398**	**0.541**	**4.9**
$\theta_{\mathrm{PL}}$	0.45	6.7	0.834	0.389	0.530	4.0

**Table 9:** Real-test ablation of the glare and chroma gates. The same nominal frozen operating threshold is used for every row; only the gate state changes. Lamp-overlap and color-overlap recall are measured on dust masks intersecting the corresponding gate support; higher is better for dust metrics and lower is better for clean metrics. The default two-gate configuration is shown in bold.

Configuration	IoU	F1	Recall	Lamp-Overlap Recall	Color-Overlap Recall	Clean FP%	Frame-FP@5%
No gates	0.389	0.521	0.695	0.728	0.706	14.7	83%
Glare gate only	0.381	0.514	0.623	0.612	0.694	8.1	60%
Chroma gate only	0.374	0.507	0.604	0.715	0.571	6.4	49%
**Both gates (default)**	**0.366**	**0.500**	**0.566**	**0.598**	**0.548**	**3.7**	**26%**

**Table 10:** Direct audit of genuine-dust suppression by the chroma gate on the eight pre-defined color-overlap dust frames. Suppression is the fraction of annotated dust pixels positive without the chroma gate but negative after it is enabled.

Frames	Mean Suppressed	Median	>10% Loss	>20% Loss	Worst Frame
8	12.4%	9.8%	5/8	2/8	31.6%

**Table 11:** Exploratory temporal-stage evaluation. Labeled IoU and clean FP use the unchanged 72 labeled target frames after temporal-buffer replay; temporal consistency and flicker use 18 held-out video windows (1350 unlabeled frames). Temporal consistency IoU compares each prediction with the preceding prediction warped by optical flow; flicker is the changed-mask area after warping. Bold values identify the better value in each metric column; higher is better for both IoU columns, whereas lower is better for clean FP, flicker, and added latency.

Configuration	Labeled IoU	Clean FP%	Temporal IoU	Flicker Area (%)	Added Latency (ms)
**Single-frame default**	**0.366**	**3.7**	0.741	4.3	**0.0**
Temporal gate (exploratory)	0.373	4.6	**0.812**	**2.9**	7.4

**Table 12:** Equal-budget student comparison after explicit BGR-to-RGB conversion and model-specific normalization. All models use the same pseudo-label split, 512 × 288 input, 60 epochs, effective batch size 8, 2040 optimizer updates, the same learning-rate grid, and seeds 11, 23 and 37. Results are mean ± standard deviation. GFLOPs count multiply-adds as two floating-point operations at 512 × 288; peak GPU memory is the maximum PyTorch allocated memory in the six-stream run. Network FPS is batch-1 forward-pass throughput; six-stream FPS and peak memory are measured with the same decode-to-mask scheduler analyzed in the subsequent runtime audit. The best numeric value in each resource or performance column is shown in bold.

Network	Params (M)	GFLOPs	Selected LR	Network FPS	Six-Stream FPS	Peak GPU (GB)	Dust IoU	F1	FP%
TinyU-Net (ours)	**0.47**	**5.1**	$2\times 10^{-3}$	**610**	**233**	**1.8**	0.398 ± 0.006	0.526 ± 0.006	4.9 ± 0.3
LR-ASPP-MobileNetV3	3.22	2.5	$1\times 10^{-3}$	259	178	2.6	**0.432 ± 0.006**	**0.563 ± 0.006**	**2.2 ± 0.2**
SegFormer-B0	3.71	4.9	$1\times 10^{-3}$	166	151	3.1	0.414 ± 0.006	0.542 ± 0.007	3.1 ± 0.3
DeepLabV3-ResNet50	41.99	96.2	$5\times 10^{-4}$	113	104	5.7	0.405 ± 0.008	0.532 ± 0.009	3.6 ± 0.3

**Table 13:** Runtime scope on RTX 4070 Ti and Intel Core i9-13900K. Timing uses FP32, batch 1, 200 warm-up iterations, 5000 measured frames, and explicit CUDA synchronization. Bold throughput values denote the two headline scopes reported in the Abstract: network-only inference and the measured six-stream aggregate.

Scope/Stage	Included Operation	Median Latency (ms)	p95 Latency (ms)	Throughput
Video decoding	H.264 decode at 2688×1520	2.63	–	–
Resize/colour conversion	2688×1520 to 512×288 RGB tensor	1.06	–	–
Host-to-device transfer	Pinned-memory tensor transfer	0.29	–	–
TinyU-Net only	Forward pass only	1.64	1.78	**610 FPS**
Upsample/threshold	Probability resize and frozen threshold	0.78	–	–
Output/queue	Mask copy and stream scheduling	0.42	–	–
Decoded-frame pipeline	All stages except video decoding	4.19	4.84	240 FPS
Full single stream	Decode to binary mask	6.82	8.31	147 FPS
Six concurrent streams	One frame per stream per scheduling cycle	25.8	29.4	**233/204 FPS aggregate (median/p95)**

**Table 14:** Agreement of the two independent primary-test annotators before adjudication. Mask intervals are session-clustered 95% confidence intervals.

Measure	Value	Analysis Set
Dust/clean condition Cohen’s κ	0.91	72 frames
Pairwise dust-mask IoU	0.79 [0.72, 0.85]	37 dust frames
Pairwise dust-mask Dice	0.88 [0.84, 0.92]	37 dust frames
Dust-area ICC(2,1)	0.94 [0.89, 0.97]	37 dust frames
Frames requiring adjudication	14/72 (19.4%)	72 frames

**Table 15:** Quantitative background consistency across the six cameras. Values are camera/session mean ± standard deviation; lower is better for NMAE and changed area, higher is better for SSIM and gradient correlation.

Comparison	Luminance NMAE	SSIM	Gradient Correlation	Area |ΔY|>20 (%)
Full background vs. independently verified clean references	0.027 ± 0.009	0.941 ± 0.018	0.901 ± 0.025	5.2 ± 1.8
Leave-one-session-out background vs. held-out median	0.031 ± 0.010	0.928 ± 0.022	0.886 ± 0.029	6.4 ± 2.1
Early-half vs. late-half background	0.041 ± 0.012	0.906 ± 0.026	0.851 ± 0.035	9.1 ± 3.0

**Table 16:** Teacher and pseudo-label sensitivity to alternative background constructions. All thresholds and gates remain frozen. Sparse and single-frame entries are mean ± standard deviation over five seeds. The nominal full temporal-median configuration is shown in bold.

Background	Dust IoU	F1	Recall	Clean FP%	Frame-FP@5%	Pseudo-Label Dice	Changed PL Pixels (%)
**Full temporal median**	**0.366**	**0.500**	**0.566**	**3.7**	**26%**	**1.000**	**0.0**
Early-half median	0.351	0.483	0.548	4.3	31%	0.914	4.8
Late-half median	0.359	0.491	0.557	4.0	29%	0.928	4.1
25% session-sparse median	0.343 ± 0.011	0.474 ± 0.012	0.539 ± 0.014	4.8 ± 0.4	34 ± 4%	0.892 ± 0.009	6.3 ± 0.5
Temporal mean	0.332	0.461	0.528	5.6	40%	0.861	8.1
Single-frame reference	0.286 ± 0.021	0.410 ± 0.024	0.472 ± 0.028	8.9 ± 1.4	57 ± 6%	0.774 ± 0.018	13.7 ± 1.3

**Table 17:** Paired, condition-stratified global-session-clustered 95% bootstrap confidence intervals on the 72-frame real set (2000 resamples). Dust-containing and clean sessions are resampled separately; all frames from an included session are retained and the same draws are used for every method. The proposed method is bold to identify the primary method rather than to claim significance in every column.

Method	Dust IoU [95% CI]	F1 [95% CI]	FP% [95% CI]
**Ours**	**0.366 [0.284, 0.451]**	**0.500 [0.397, 0.594]**	**3.7 [2.2, 5.8]**
DCP’09	0.358 [0.276, 0.442]	0.490 [0.386, 0.585]	14.8 [11.3, 18.8]
Grounded-SAM2’24	0.280 [0.211, 0.354]	0.400 [0.326, 0.477]	59.9 [49.0, 69.8]
Wavelet’06	0.274 [0.205, 0.346]	0.410 [0.333, 0.487]	22.6 [17.0, 28.8]
PaDiM’20	0.247 [0.181, 0.316]	0.365 [0.287, 0.444]	24.9 [18.4, 31.9]
PatchCore’22	0.263 [0.195, 0.334]	0.389 [0.310, 0.467]	19.7 [14.2, 25.9]
SimpleNet’23	0.281 [0.211, 0.353]	0.406 [0.326, 0.486]	16.8 [11.9, 22.3]
EfficientAD	0.294 [0.223, 0.367]	0.428 [0.346, 0.509]	13.9 [9.5, 19.0]

**Table 18:** Synthetic-calibration sensitivity to opacity, airlight, morphology, and illumination interaction. Each row selects its threshold only on the stated synthetic variant and applies it unchanged to the real set. The real-oracle value is reported only in text as a post hoc diagnostic. The nominal generator row is shown in bold.

Synthetic Calibration Variant	Selected τ	Synthetic IoU	Real IoU	Real FP%
**Nominal generator**	**0.375**	**0.603**	**0.366**	**3.7**
20% lower opacity	0.350	0.584	0.359	4.3
20% higher opacity	0.400	0.611	0.362	3.4
Warm airlight perturbation	0.375	0.596	0.364	3.8
Cool airlight perturbation	0.375	0.592	0.361	3.9
Smaller/sparser plume morphology	0.350	0.570	0.352	4.5
Larger/denser plume morphology	0.400	0.618	0.358	3.3
Lamp-adjacent plume placement	0.400	0.588	0.357	3.6
Low-illumination plume placement	0.350	0.574	0.354	4.2
Spatially varying airlight	0.375	0.597	0.363	3.8

**Table 19:** Leave-one-camera-out synthetic-threshold diagnostic. The held-out camera contributes no synthetic calibration sample but retains its own unlabelled background model. The aggregate row is shown in bold.

Held-Out Camera	Selected τ	Dust IoU	F1	FP%
Cam01	0.375	0.142	0.249	1.8
Cam02	0.375	0.303	0.452	1.7
Cam03	0.400	0.708	0.829	6.2
Cam04	0.375	0.428	0.568	2.7
Cam05	0.400	0.165	0.269	6.1
Cam06	0.375	0.377	0.538	3.4
**Aggregate**	–	**0.356**	**0.489**	**3.9**

**Table 20:** Per-camera breakdown of the proposed method. Cam01–Cam06 are anonymized aliases and do not denote camera-held-out test partitions. “Dust Frames” and “Clean Frames” are frame counts.

Camera	Dust Frames	Clean Frames	IoU	F1	FP%
Cam01	6	5	0.146	0.253	1.6
Cam02	5	6	0.311	0.460	1.5
Cam03	4	7	0.724	0.840	5.9
Cam04	11	5	0.439	0.579	2.4
Cam05	4	8	0.172	0.276	5.8
Cam06	7	4	0.386	0.549	3.1

**Table 21:** Segmentation performance as a function of annotated dust-area ratio $\rho_{G}=∥G∥/\left(HW\right)$. Bins were fixed before detector scoring; counts sum to the 37 positive frames, and *n* denotes the number of frames in each bin.

Dust-Area Ratio	*n*	IoU	F1	Recall
0–3%	6	0.121	0.208	0.286
3–7%	8	0.203	0.328	0.388
7–12%	8	0.298	0.426	0.492
12–20%	7	0.451	0.596	0.646
>20%	8	0.596	0.722	0.784

**Table 22:** Frame-level false-positive response on 35 clean frames after fixed baseline-normal score calibration. Lower values are better; the lowest value in each column is shown in bold.

Method	Mean FP%	Frame-FP@1%	Frame-FP@5%	Frame-FP@10%
Ours	**3.7**	**77%**	**26%**	**6%**
DCP	14.8	100%	97%	86%
Grounded-SAM2	59.9	100%	100%	100%
Wavelet	22.6	97%	83%	63%
PaDiM	24.9	100%	91%	77%
PatchCore	19.7	100%	86%	69%
SimpleNet	16.8	97%	80%	57%
EfficientAD	13.9	94%	71%	43%

**Table 23:** Performance on held-out dust-only and spray-only conditions under the same frozen operating settings used for the primary real evaluation. Up arrows indicate that higher values are better for dust metrics; down arrows indicate that lower values are better for spray metrics. The best value in each column is shown in bold. Session-clustered 95% intervals for the principal DustVeil and DCP outcomes are reported in the text and Supplementary Table S12.

Method	Dust IoU ↑	Dust F1 ↑	Dust Recall ↑	Spray Mean Area (%) ↓	Spray-Mask Response (%) ↓	Spray Frame-FP@5% (%) ↓
Ours	**0.371**	**0.508**	0.579	**4.1**	**12.6**	**22.2**
DCP’09	0.362	0.497	**0.601**	15.9	44.8	86.1
Grounded-SAM2’24	0.287	0.408	0.486	31.7	57.3	97.2
Wavelet’06	0.274	0.410	0.565	28.7	51.4	83.3
PaDiM’20	0.255	0.377	0.504	22.8	48.2	77.8
PatchCore’22	0.270	0.398	0.529	18.9	42.6	72.2
SimpleNet’23	0.286	0.412	0.548	16.4	38.1	66.7
EfficientAD	0.298	0.429	0.561	14.5	34.7	61.1

## Data Availability

Supplementary Data S1 contains the anonymized clip/session-role manifest; Supplementary Audit S1 provides the manifest-validation report; Supplementary Tables S1–S23 provide the derived audits; and Supplementary Code S1 contains the source code, pinned configuration files, and commands used to generate the reported audit tables. The row-level manifest and source imagery for the held-out water-spray specificity analysis are controlled by an industrial engineering collaborator and are not included in the public Supplementary File. Access requests may be directed to the corresponding author and will be considered only with authorization from the data owner, mine-operator approval, and a data-use agreement. Raw underground video, the private file-name mapping, and the 72-frame image pixels are governed by the same restrictions; anonymized indices and annotations for the primary corpus are documented in Supplementary Data S1.

## Supplementary Materials

The following supporting information can be downloaded at: https://www.mdpi.com/article/10.3390/s26175635/s1, Supplementary Data S1 provides the anonymized corpus manifest; Supplementary Audit S1 provides the manifest-validation report; Supplementary Tables S1–S19 contain the previous allocation, annotation, robustness, threshold, ablation, and efficiency audits; Supplementary Tables S20–S23 add the fixed-versus-per-image score-calibration audit, corrected student-preprocessing audit, reproducibility-command matrix, and returned-output/model-I/O specification. Supplementary Code S1 contains executable scripts, frozen configurations, expected CSV schemas, and smoke tests for baseline calibration, gate/temporal ablation, and timing.
