# Supplementary material for: DustVeil: Label-Free Real-Time Detection of Airborne Coal-Mine Dust in Camera Streams via Physically-Grounded Multi-Cue Fusion and Knowledge Distillation
Source: Sensors (Basel). 2026 Sep 4;26(17):5635. doi: 10.3390/s26175635 (PMC13568155; doi:10.3390/s26175635)
Supplement: Supplementary file 1 [file sensors-26-05635-s001.zip › DustVeil_Supplementary_Audits_R1-R4_NO_WATERMARK.pdf]

Supplementary Manifest and Leakage Audit

Manuscript ID: sensors-4493537

DustVeil: Label-Free Real-Time Detection of Airborne Coal-Mine Dust in Camera Streams

Row-level identifiers are anonymized; the audit retains the grouping relations required for leakage verification.

Data S1: Anonymized Row-Level Manifest

The accompanying CSV contains 493 anonymized clip rows from 107 global recording sessions and six cameras. Each clip and each global session belongs to exactly one role. The recorded SHA-256 hash of the anonymized CSV is:

```
429a6ebf8ad52955ce6375e87b9324dca54e9d76ed0ea03d11ad8097d0cab987
```

The coarse condition field was assigned retrospectively after the six-role manifest was locked. It is used for descriptive reporting only; the predefined role-eligibility rules, including clean-footage requirements for background, baseline-normal and synthetic-base clips, remain unchanged.

Supplementary Table S1: Per-Camera Role Allocation

Camera	Background	Pseudo train	Pseudo val	Baseline normal	Synthetic	Real test	Total
C1	25	15	4	25	2	12	83
C2	24	15	4	25	2	12	82
C3	24	15	4	25	2	12	82
C4	24	15	4	25	2	12	82
C5	24	15	4	25	2	12	82
C6	24	15	4	25	2	12	82
Total	145	90	24	150	12	72	493

Table 1: Clip counts by anonymized camera and exclusive manifest role.

Supplementary Table S2: Role-by-Condition Composition

Role	Clean	Dust-dominant	Spray-dominant	Mixed	Uncertain	Total
background	145	0	0	0	0	145
pseudo_train	20	45	15	8	2	90
pseudo_val	5	12	4	2	1	24
baseline_normal	150	0	0	0	0	150
synthetic	12	0	0	0	0	12
real_test	35	37	0	0	0	72
Total	367	94	19	10	3	493

Table 2: Coarse visual-condition counts. Tags were assigned after locking the six-role split and are used only for descriptive reporting.

Supplementary Audit S1: Validator Results

Audit item	Observed	Required
Manifest rows	493	493
Unique clip identifiers	493	493
Unique global sessions	107	107
Unique camera identifiers	6	6
Missing camera identifiers	0	0
Missing role values	0	0
Missing global session identifiers	0	0
Duplicate clip rows	0	0
Clips assigned to more than one role	0	0
Global sessions assigned to more than one role	0	0
Real-test clip overlap with other roles	0	0
Real-test session overlap with other roles	0	0
Specificity clip overlap with original corpus	0	0
Specificity session overlap with original corpus	0	0
Sum of role-specific clip counts	493	493

Manifest SHA-256: `429a6ebf8ad52955ce6375e87b9324dca54e9d76ed0ea03d11ad8097d0cab987`

Supplementary Quantitative Audits for Reviewer 1

Manuscript ID: sensors-4493537

Table S3. Background quality and chronological drift

Comparison	NMAE	SSIM	Gradient correlation	Changed area (%)
Verified clean references (60 frames)	0.027 ± 0.009	0.941 ± 0.018	0.901 ± 0.025	5.2 ± 1.8
Leave-one-session-out consistency	0.031 ± 0.010	0.928 ± 0.022	0.886 ± 0.029	6.4 ± 2.1
Early versus late sessions (14.0 [7.0, 21.0] days)	0.041 ± 0.012	0.906 ± 0.026	0.851 ± 0.035	9.1 ± 3.0

Table S4. Sensitivity to alternative background construction

Background	IoU	F1	Recall	Clean FP	frame-FP@5%	PL Dice	PL change
Full median	0.366	0.500	0.566	3.7	26	1.000	0.0
Early half	0.351	0.483	0.548	4.3	31	0.914	4.8
Late half	0.359	0.491	0.557	4.0	29	0.928	4.1
25% sparse	0.343 ± 0.011	0.474 ± 0.012	0.539 ± 0.014	4.8 ± 0.4	34 ± 4	0.892 ± 0.009	6.3 ± 0.5
Temporal mean	0.332	0.461	0.528	5.6	40	0.861	8.1
Single frame	0.286 ± 0.021	0.410 ± 0.024	0.472 ± 0.028	8.9 ± 1.4	57 ± 6	0.774 ± 0.018	13.7 ± 1.3

Table S5. Annotation reliability

Audit item	Result
Condition-label Cohen's κ	0.91
Pairwise mask IoU, clustered 95% CI	0.79 [0.72, 0.85]
Pairwise mask Dice, clustered 95% CI	0.88 [0.84, 0.92]
Annotated-area ICC, 95% CI	0.94 [0.89, 0.97]
Frames requiring adjudication	14/72 (19.4%)
DustVeil IoU: A/B/consensus	0.354/0.361/0.366
DCP IoU: A/B/consensus	0.347/0.353/0.358

Table S6. Condition-stratified global-session-clustered bootstrap

Method	IoU [95% CI]	F1 [95% CI]	Clean FP [95% CI]
DustVeil	0.366 [0.284, 0.451]	0.500 [0.397, 0.594]	3.7 [2.2, 5.8]
DCP	0.358 [0.276, 0.442]	0.490 [0.386, 0.585]	14.8 [11.3, 18.8]
Grounded-SAM2	0.280 [0.211, 0.354]	0.400 [0.326, 0.477]	59.9 [49.0, 69.8]
Wavelet	0.274 [0.205, 0.346]	0.410 [0.333, 0.487]	22.6 [17.0, 28.8]
PaDiM	0.247 [0.181, 0.316]	0.365 [0.287, 0.444]	24.9 [18.4, 31.9]
PatchCore	0.263 [0.195, 0.334]	0.389 [0.310, 0.467]	19.7 [14.2, 25.9]
SimpleNet	0.281 [0.211, 0.353]	0.406 [0.326, 0.486]	16.8 [11.9, 22.3]
EfficientAD reference implementation	0.294 [0.223, 0.367]	0.428 [0.346, 0.509]	13.9 [9.5, 19.0]
DustVeil minus DCP	0.008 [-0.021, 0.038]	0.010 [-0.020, 0.041]	-11.1 [-14.4, -8.1]

Table S7. Threshold-transfer diagnostics

Variant	Threshold	Synthetic IoU	Real IoU	Real clean FP
Nominal	0.375	0.603	0.366	3.7
Low opacity	0.350	0.584	0.359	4.3
High opacity	0.400	0.611	0.362	3.4
Warm airlight	0.375	0.596	0.364	3.8
Cool airlight	0.375	0.592	0.361	3.9
Sparse plume	0.350	0.570	0.352	4.5
Dense plume	0.400	0.618	0.358	3.3
Lamp-adjacent plume	0.400	0.588	0.357	3.6
Low-illumination plume	0.350	0.574	0.354	4.2
Spatially varying airlight	0.375	0.597	0.363	3.8

Table S8. Baseline implementation and output-level protocol

Method	Status	Fitting/input	Prompt or score	Output handling / threshold
Wavelet	Reference adaptation	RGB test frame	Three-level Haar motion/texture score	Score clipped to [0,1]; 960×543; no morphology; synthetic-selected threshold
DCP	Equation-level reimplementation	RGB + camera background	15×15 dark-channel/airlight score	Score clipped to [0,1]; 960×543; no morphology; synthetic-selected threshold
PaDiM	Reference adaptation	50 normal frames/camera; 256×256 ImageNet input	Mahalanobis anomaly score	Per-camera 1st/99th normal-score percentile normalization; no morphology; synthetic-selected threshold
PatchCore	Author-reference adapter	50 normal frames/camera; 256×256 ImageNet input	Nearest-neighbour anomaly score	Per-camera 1st/99th normal-score percentile normalization; no morphology; synthetic-selected threshold
SimpleNet	Author-reference adapter	50 normal frames/camera; 256×256 ImageNet input	Learned anomaly score	Per-camera 1st/99th normal-score percentile normalization; no morphology; synthetic-selected threshold
EfficientAD	Reference implementation of published PDN/student–teacher/autoencoder design	50 normal frames/camera; model-specific RGB normalization	Published anomaly score	Fixed per-camera 1st/99th baseline-normal calibration; no morphology; synthetic-selected threshold
Grounded-SAM2	Official pretrained models; zero-shot	Original-aspect-ratio RGB frame	"coal dust. airborne dust cloud."; box/text .20/.18	Union selected masks; fixed thresholds; no prompt editing or morphology

Table S9. Complete gate ablation

Variant	IoU	F1	Recall	Lamp recall	Colour recall	Clean FP	frame-FP@5%
No gates	0.389	0.521	0.695	0.728	0.706	14.7	83
Glare only	0.381	0.514	0.623	0.612	0.694	8.1	60
Chroma only	0.374	0.507	0.604	0.715	0.571	6.4	49
Both gates	0.366	0.500	0.566	0.598	0.548	3.7	26

Table S10. Temporal stability and latency

Configuration	Labelled IoU	Clean FP	Consistency IoU	Flicker	Added ms	Warm-up ms
Single-frame default	0.366	3.7	0.741	4.3	0.0	0
Optional temporal gate	0.373	4.6	0.812	2.9	7.4	160

Table S11. Equal-budget students and runtime

Student	Params	LR	Updates	Seeds	Net FPS	6-stream FPS	Peak GB	IoU	F1	Clean FP
TinyU-Net	.47	2e-3	2040	3	610	233	1.8	0.398 ± 0.006	0.526 ± 0.006	4.9 ± 0.3
LR-ASPP-MobileNetV3	3.22	1e-3	2040	3	259	178	2.6	0.432 ± 0.006	0.563 ± 0.006	2.2 ± 0.2
SegFormer-B0	3.71	1e-3	2040	3	166	151	3.1	0.414 ± 0.006	0.542 ± 0.007	3.1 ± 0.3
DeepLabV3-ResNet50	41.99	5e-4	2040	3	113	104	5.7	0.405 ± 0.008	0.532 ± 0.009	3.6 ± 0.3

Runtime scope	Median ms	p95 ms	Throughput	Included operation
TinyU-Net only	1.64	1.78	610 FPS	Forward pass only
Decoded-frame pipeline	4.19	4.84	240 FPS	Preprocess, transfer, inference, postprocess, output
Full single stream	6.82	8.31	147 FPS	H.264 decode to binary mask
Six-stream scheduling cycle	25.8	29.4	233 FPS median; 204 FPS p95	One frame from each of six streams per cycle

Table S12. Water-spray specificity confidence intervals

Outcome	DustVeil	DCP
Dust-only IoU [95% CI]	0.371 [0.305, 0.438]	0.362 [0.294, 0.431]
Spray mean area [95% CI]	4.1 [2.7, 5.9]	15.9 [12.0, 20.5]
Spray-mask response [95% CI]	12.6 [8.1, 18.4]	44.8 [36.3, 53.4]
Spray frame-FP@5% [95% CI]	22.2 [8.3, 41.7]	86.1 [66.7, 100.0]

DustVeil: Reviewer 2 Quantitative Audit Tables

Supplementary Tables S13–S19

These tables provide the detailed values supporting the Reviewer 2 analyses. All cue and operating thresholds are selected without using real-test masks for model selection; the real masks are used only for the reported post-hoc evaluation.

Table S13. Complete cue-combination ablation

Configuration	Synthetic-selected τ	Real IoU	Real F1	Recall	Clean FP (%)
C1: veiling	0.325	0.274	0.392	0.515	11.8
C2: texture decay	0.350	0.301	0.421	0.548	17.6
C3: dark channel	0.400	0.358	0.490	0.638	14.8
C1+C2	0.350	0.329	0.454	0.598	15.9
C1+C3	0.375	0.376	0.516	0.674	17.2
C2+C3	0.375	0.369	0.508	0.661	18.5
C1+C2+C3, no gates	0.375	0.389	0.521	0.695	14.7

Among the ungated cue sets, C1+C2+C3 gives the highest real-test IoU (0.389), while the full three-cue response reaches recall 0.695 before suppression. The coordinated cue and gate ablations therefore show that the final operating point is not obtained by an unreported sequence of tuning decisions: pairwise cue complementarity is measured first, and the two gates then trade recall for a substantially lower clean-frame false-positive area.

Table S14. Sensitivity to τ_m and θ_{PL}

Parameter	Value	Pseudo FG (%)	Pseudo-val Dice	Real IoU	Real F1	Recall	Clean FP (%)
τ_m	0.250	–	–	0.331	0.462	0.688	8.9
τ_m	0.300	–	–	0.352	0.484	0.634	5.8
τ_m	0.325	–	–	0.360	0.493	0.611	4.7
τ_m	0.350	–	–	0.364	0.498	0.589	4.1
τ_m (nominal)	0.375	–	–	0.366	0.500	0.566	3.7
τ_m	0.400	–	–	0.364	0.497	0.541	3.2
τ_m	0.425	–	–	0.359	0.491	0.516	2.8
τ_m	0.450	–	–	0.351	0.482	0.488	2.4
τ_m	0.500	–	–	0.331	0.458	0.431	1.9
θ_{PL}	0.30	13.8	0.801	0.382	0.515	–	7.1
θ_{PL}	0.35	10.9	0.829	0.393	0.529	–	5.8
θ_{PL} (nominal)	0.40	8.6	0.842	0.398	0.541	–	4.9
θ_{PL}	0.45	6.7	0.834	0.389	0.530	–	4.0
θ_{PL}	0.50	5.1	0.812	0.368	0.506	–	3.4

The teacher operating threshold has a broad performance region: thresholds 0.325–0.425 remain within 0.007 IoU of the best observed value, while the nominal synthetic-selected value 0.375 yields the maximum IoU/FP compromise. The pseudo-label threshold also shows a stable central region (0.35–0.45); very low values increase foreground noise and false positives, whereas very high values remove faint plume supervision.

Table S15. Full clean-frame predicted-area exceedance curve

Predicted area threshold (%)	DustVeil exceedance (%)	DCP exceedance (%)
0.5	88.6	100.0
1	77.1	100.0
2	51.4	97.1
3	40.0	97.1
5	25.7	97.1
7.5	14.3	94.3
10	5.7	85.7
15	2.9	62.9
20	0.0	40.0
Normalized area under exceedance curve	16.5	78.6

Table S16. IoU/F1 as a function of annotated dust-area ratio

Dust-area ratio	Frames	IoU	F1	Recall
0–3%	6	0.121	0.208	0.286
3–7%	8	0.203	0.328	0.388
7–12%	8	0.298	0.426	0.492
12–20%	7	0.451	0.596	0.646
>20%	8	0.596	0.722	0.784

Session-bootstrap Spearman association between annotated dust-area ratio and IoU: 0.68 [0.47, 0.81].

Table S17. Genuine-dust suppression by the chroma gate

Subset frames	Mean suppressed GT dust (%)	Median (%)	Frames > 10%	Frames > 20%	Worst frame (%)
8	12.4	9.8	5/8	2/8	31.6

On the 8 colour-overlap dust frames, the chroma gate removes a mean 12.4% of annotated dust pixels (median 9.8%); 5/8 frames lose more than 10% and 2/8 lose more than 20%. The worst observed frame loses 31.6%, quantifying the cost behind the lower colour-overlap recall in the gate ablation.

Table S18. Training augmentation audit

Policy	Pseudo-val Dice	Real IoU	Real F1	Clean FP (%)
No augmentation	0.817	0.386	0.523	5.7
Standard augmentation	0.842	0.398	0.541	4.9

The student uses moderate geometry-preserving and photometric augmentation. Relative to no augmentation, the standard policy changes pseudo-validation Dice from 0.817 to 0.842, real IoU from 0.386 to 0.398, and clean FP from 5.7% to 4.9%.

Table S19. Expanded student efficiency audit

Network	Parameters (M)	GFLOPs	Network FPS	Six-stream FPS	Peak GPU memory (GB)
TinyU-Net	0.47	5.1	610	233	1.8
LR-ASPP-MobileNetV3	3.22	2.5	259	178	2.6
SegFormer-B0	3.71	4.9	166	151	3.1
DeepLabV3-ResNet50	41.99	96.2	113	104	5.7

Efficiency is now reported using parameters, multiply-add-derived GFLOPs, network-only FPS, six-stream throughput and peak allocated GPU memory at the common 512×288 input. TinyU-Net uses 5.1 GFLOPs and 1.8 GB peak memory; the comparison no longer equates parameter count alone with deployment cost.

DustVeil Supplementary Reviewer 3–4 Implementation and Reproducibility Audits

Table S20. Fixed score-calibration audit

The legacy implementation normalized each baseline map independently. The revised implementation fits one 1st–99th percentile affine mapping per method/camera from baseline-normal frames and freezes it. Values below are clean-frame false-positive area percentages.

Method	Legacy per-image min–max	Fixed normal calibration
Wavelet	61.2	22.6
PaDiM	90.2	24.9
PatchCore	96.9	19.7
SimpleNet	99.8	16.8
EfficientAD	78.0	13.9

Table S21. Student input-preprocessing correction

All OpenCV frames are converted BGR to RGB. Pretrained convolutional backbones use ImageNet mean/standard deviation, and SegFormer uses its model processor.

Network	Revised preprocessing	Legacy IoU	Corrected IoU
TinyU-Net	RGB/[255], trained from scratch	0.398 ± 0.006	0.398 ± 0.006
LR-ASPP-MobileNetV3	RGB + ImageNet mean/std	0.421 ± 0.005	0.432 ± 0.006
SegFormer-B0	RGB + model processor	0.392 ± 0.007	0.414 ± 0.006
DeepLabV3-ResNet50	RGB + ImageNet mean/std	0.374 ± 0.009	0.405 ± 0.008

Table S22. Reproduction-command coverage

Reported result	Executable command	Output
Fixed score calibration	`audit_baseline_normalization.py`	percentile record and S20 audit
Student preprocessing	`audit_student_preprocessing.py`	corrected input audit and S21
Gate ablation	`reproduce_gate_ablation.py`	four gate configurations with IoU/F1/recall/FP
Temporal audit	`reproduce_temporal_ablation.py`	single-frame/temporal consistency, flicker and latency
Network timing	`benchmark_tiny_unet.py`	synchronized network-only latency and FPS

Table S23. Returned outputs and tensor interfaces

Component	Input	Output	Interpretation
Teacher cues	543×960×3 RGB	three 543×960 maps	software-derived visual evidence
Fusion/gates	cue and gate maps	543×960, [0,1]	dimensionless visual response
Binary output	response map	543×960 mask	frozen-threshold plume region
TinyU-Net	1×3×288×512	1×1×288×512	student probability
Area ratio	binary mask	scalar [0,1]	image fraction; not concentration or hazard
